# Development of an interprofessional education program for aspiration pneumonia care: an action research study

**DOI:** 10.1007/s41999-026-01490-2

**Published:** 2026-08-10

**Authors:** Tadayuki Hashimoto, Yuki Yoshimatsu, Shinichi Matsumoto, Takayuki Ando, Kiichi Enomoto, Yasuhisa Fukuzoe, Keiko Hirose, Haruki Imura, Tomohiro Matsumoto, Taiju Miyagami, Yoichi Ohtake, Mamiko Ukai, Toru Morikawa

**Affiliations:** 1 Department of Emergency Medicine, Brigham and Women’s Hospital, 75 Francis St, Boston, MA 02115 USA; 2 Department of General Medicine, Osaka Medical and Pharmaceutical University, Osaka, Japan; 3 Ageing and Health, Guy’s and St Thomas’ NHS Foundation Trust, London, UK; 4 Centre for Exercise Activity and Rehabilitation, University of Greenwich, London, UK; 5 Yushoukai Zaitaku Clinic Katsushika, Tokyo, Japan; 6 Center for General Medicine Education, Keio University School of Medicine, Tokyo, Japan; 7 Department of Pharmacy, Nerima Hikarigaoka Hospital, Tokyo, Japan; 8 Department of Nursing, Kawanishi City Medical Center, Hyogo, Japan; 9 Department of Nutrition Management, Nerima Hikarigaoka Hospital, Tokyo, Japan; 10 Department of Infectious Diseases, Rakuwakai Otowa Hospital, Kyoto, Japan; 11 Department of Internal Medicine, Uenohara City Hospital, Yamanashi, Japan; 12 Department of General Medicine, Juntendo University Faculty of Medicine, Tokyo, Japan; 13 Division of Hospital Medicine, University of Colorado, Aurora, CO USA; 14 Takeo Clinic/Body Mind Clinic, Osaka, Japan; 15 Ouchino Clinic Medical Corporation, Tokyo, Japan; 16 Department of General Medicine, Nara City Hospital, Nara, Japan; 17 Department of Data Science, Hyogo Medical University, Hyogo, Japan

**Keywords:** Pneumonia, Aspiration, Interprofessional education, Patient care team, Action research, Curriculum development, Kern’s six-step model

## Abstract

**Introduction/aim:**

Aspiration pneumonia has evolved, with demographics shifting to predominantly frail older adults. However, despite the high healthcare impact, there is no established guideline or curriculum for the education and training of healthcare professionals in managing aspiration pneumonia in older adults. This study aimed to explore optimal educational interventions to improve holistic management of aspiration pneumonia in older adults, and to identify barriers and facilitators towards developing multidisciplinary teams specialized in this area.

**Methods:**

We conducted a multi-year action research study from 2019 to 2024 using Kern’s six-step model across three annual cycles: exploratory (2020), prototyping (2021), and refinement (2022) phases. Participating hospitals across Japan recruited interprofessional teams consisting of physicians, dentists, nurses, pharmacists, physical, occupational, and speech-language therapists, dietitians, social workers, and care workers. The educational intervention combined streamlined pre-learning modules with interactive workshops featuring case-based simulation. Primary outcomes included participant satisfaction and establishment of a new multidisciplinary team at 6-month follow-up. We integrated quantitative metrics and qualitative thematic analysis to analyze data.

**Results:**

A total of 131 participants from 32 hospitals participated. Overall satisfaction scores remained consistently high: 4.4 ± 0.6 (2020), 4.5 ± 0.5 (2021), and 4.5 ± 0.6 (2022) out of 5.0. Pre-learning completion rates improved substantially from 84.4 to 96.1% following weekly reminders and mobile optimization. At 6-month follow-up, 37.5% (3/8) of teams without pre-existing multidisciplinary teams established new teams, while all other teams reported enhanced interprofessional activities and behavioral changes.

**Conclusion:**

Through iterative refinement in a collaborative practice paradigm, we demonstrated the critical importance of interprofessional collaboration in the management of older adults with aspiration pneumonia.

**Supplementary Information:**

The online version contains supplementary material available at https://doi.org/10.1007/s41999-026-01490-2.

## Key summary points

**Aim:**

To address the growing global burden of aspiration pneumonia in older adults with frailty, a major cause of recurrent hospitalization, mortality, and healthcare system strain, by developing and evaluating an interprofessional education program to improve collaborative care.

**Findings:**

This multi-year action research study demonstrates that structured interprofessional education can strengthen collaborative practice, facilitate the formation of new multidisciplinary teams, and promote sustained behavioral and organizational changes in aspiration pneumonia care. In addition, the study clarifies how such programs can be systematically structured and implemented across diverse clinical settings, for example through learner-friendly, streamlined pre-learning modules combined with interactive, case-based simulation workshops.

**Message:**

Although interprofessional collaboration has long been considered essential for aspiration pneumonia care, this study shows that such collaboration can be systematically strengthened and sustained through targeted education.

**Supplementary Information:**

The online version contains supplementary material available at https://doi.org/10.1007/s41999-026-01490-2.

## Introduction

Globally, aspiration pneumonia is now a serious socioeconomic challenge, responsible for a significant proportion (up to 90%) of pneumonia in the older population [1]. Aspiration pneumonia is highly associated with recurrent and prolonged hospital admissions and increased mortality [2–4]. It significantly impacts the quality of life of patients and caregivers [5–7], and leads to a high burden on healthcare professionals due to its complexity [8, 9]. Pneumonia is the most common cause of death from infectious diseases, with an overall incidence rate among adults of 2–5 cases per 1,000 person-years and 1.13 million deaths in those over 70 years in 2017 [3]. In older adults, community-associated pneumonia related with aspiration has significantly worse in-hospital mortality compared with those with non-aspiration pneumonia (27.6 vs. 19.0%) and 1-year mortality (64.2 vs. 53.2%) [4]. Therefore, fundamental measures are indispensable to facilitate better management of aspiration pneumonia in older adults. A paradigm shift has been suggested in the diagnosis of pneumonia in older adults, where clinicians focus not on differentiating pneumonia between aspiration and non-aspiration, but assessing for aspiration in all older adults with pneumonia [10], under the concept of ‘frailty-associated pneumonia’ [11].

Despite the increasingly heavy burden of aspiration pneumonia in older adults on healthcare, and a surge in research interest [12], there is still no established guideline on its management. Furthermore, there is no curriculum for the education and training of healthcare professionals. Patients with aspiration pneumonia often present with frailty and multimorbidity, requiring prolonged and recurrent hospital admissions with complex decision-making and end-of-life care. There is also a high socioeconomic impact, warranting structured training for healthcare professionals to improve patient care. Current medical education, predominantly focused on curative pharmacotherapy, often fails to address the critical need for multidisciplinary team (MDT) approaches and non-curative management strategies [8, 9]. With a mission to tackle this gap in clinical demand and available guidance, a multidisciplinary organization was developed in 2019 to deliver effective evidence-based training in this area, namely the Japan Aspiration pneumonia inter-Professional team Educational Program (JAPEP). JAPEP has been developing and delivering training programs for healthcare professionals to facilitate MDT involvement in the management of aspiration pneumonia in older adults.

The aim of this qualitative study was to explore the educational interventions necessary to improve the holistic management of aspiration pneumonia in older adults. The objectives were to identify what factors are necessary for a satisfactory and effective online MDT training, and to identify the barriers and facilitators towards developing an MDT specialized in the management of aspiration pneumonia in older adults.

## Methods

### Action research

We undertook an action research, which “is an iterative process in which researchers and practitioners act together in the context of an identified problem to discover and effect positive change.” [13] The methodology follows cyclical phases of ‘planning’, ‘acting’, ‘observing’, and ‘reflecting’, with each cycle informing refinements to subsequent interventions [14, 15]. This approach is well-suited for healthcare quality improvement initiatives, allowing real-time adaptation based on participant feedback while maintaining scientific rigor.

Throughout our project period (2019–2024), we implemented three action research cycles annually from 2020 to 2022: (1) planning educational interventions as ‘planning’; (2) implementing the program as ‘acting’; (3) observing outcomes through quantitative and qualitative data collection as ‘observing’; and (4) reflecting on results with participants as ‘reflecting’. We characterized the three cycles as exploratory, prototyping, and refinement phases to reflect the progressive nature of program evolution.

This study drew upon best practices for reporting action research [16] and quality improvement research (SQUIRE 2.0) [17, 18].

### Kern’s six-step model for curriculum development

We employed Kern’s six-step approach as the systematic framework for program design: (1) problem identification and general needs assessment; (2) targeted needs assessment; (3) goals and objectives; (4) educational strategies; (5) implementation; and (6) evaluation and feedback [19]. This model’s iterative nature aligned well with our action research methodology, allowing systematic refinement across cycles based on observed outcomes and stakeholder feedback.

### Theoretical framework

This study was grounded in pragmatist epistemology, prioritizing practical problem-solving while maintaining systematic inquiry. We drew on sociocultural learning theories, particularly Communities of Practice (CoP) [20] and Situated Learning Theory [21], which conceptualize learning as participation in social practices. We also applied Transformative Learning Theory [22] to understand how our assumptions about the problem evolved through critical reflection on participant feedback.

### Study design

This multi-year action research study was conducted from 2019 to 2024 in three stages (Table 1). The conceptualization stage (2019) involved organizer team formation, needs assessment and initial program design.

**Table 1:** Overview of study stages, phases, and participants

Stage	Year	Phase (action research cycle)	Number of participants (hospitals)	Participant composition	Main activities
Conceptualization	2019	-	-	-	Team building, KJ method to identify 7 domains and 22 competencies
Program development (action research phase)	2020	Exploratory (Cycle 1)	35 (N/A)	35 MD	Physician-only webinar (7 domains 22 competencies), testing initial approach, discovery of need for MDTs
	2021	Prototyping (Cycle 2)	45 (6)	6 MD, 2 DDS, 10 RN, 5 Ph, 17 Th, 3 RD 2 SW	MDTs introduced, flipped classroom format, redesign of competencies (7 domains, 23 competencies) based on webinar feedback, introduction of educational framework, new educational strategies
	2022	Refinement (Cycle 3)	51 (8)	9 MD, 1 DDS, 9 RN, 1 NP, 4 Ph, 13 PT/OT, 1 SLT, 7 RD, 3 DH, 3 SW	Case-based simulation, streamlined pre-learning, scoping review to refine competencies (12 domains), optimized final version, expanded multi-professional faculty
Implementation	2023	-	52 (7)	8 MD, 16 RN, 3 Ph, 18 Th, 4 RD, 2 DH, 1 SW	Finalized program evaluation at multiple institutions, 6-month follow-up assessment
	2024	-	74 (10)	10 MD, 4 DDS, 20 RN, 5 Ph, 18 Th, 8 RD, 5 DH, 3 SW, 1 CW	Finalized program evaluation at multiple institutions, 6-month follow-up assessment

CW, care worker; DDS, dentist; DH, dental hygienist; KJ method, Kawakita Jiro method; MD, physician; NP, nurse practitioner; OT, occupational therapist; Ph, pharmacist; PT, physical therapist; RD, dietitian; RN, nurse; SLT, speech and language therapist; SW, social worker; Th, therapist (PT/OT/ST combined)

The program development stage (2020–2022) consisted of three annual action research cycles—exploratory (2020), prototyping (2021), and refinement (2022) phases—during which the program was iteratively tested and refined. The exploratory phase tested initial approaches with physicians. The prototyping phase introduced MDTs and flipped classroom (a pedagogical method in which learners prepare for scheduled teaching sessions by familiarizing themselves with the relevant material in advance of a classroom session) [23]. The refinement phase optimized the program to create the final version.

The implementation stage (2023–2024) evaluated the finalized program at multiple institutions.

### Setting and participants

Participating hospitals were across Japan (including acute care, chronic care, and rehabilitation hospitals), recruited through the Japan Primary Care Association mailing list. Eligibility criteria included presence of inpatient wards caring for older patients with aspiration pneumonia and willingness to dedicate staff time for educational activities.

The exploratory phase (2020) included physicians only. From the prototyping phase (2021) onward, we required hospital-based MDTs. Team eligibility criteria included: (1) one team per hospital, (2) mandatory participation of at least one physician or dentist, (3) minimum of three professions represented, (4) team size of 4–8 members, and (5) eligible professions included physicians, dentists, nurses, pharmacists, physical/occupational/speech and language therapists, dietitians, medical social workers, care workers, and dental hygienists. Participation was voluntary with no exclusion criteria based on experience or prior training.

### Data collection

We collected multiple data sources to evaluate program implementation and participant engagement:

*Participant characteristics* We collected demographic data (professional role, years of clinical experience).

*Pre-learning engagement* We tracked completion rates of streamlined pre-learning modules prior to workshop attendance. During the prototyping phase, we collected aggregate, anonymized platform analytics from the Moodle learning management system to identify device types used to access learning materials. No individual-level tracking or personally identifiable information was collected or processed. This data collection fell within the scope of the original IRB approval (R2.10-1) and did not require additional ethical review.

*Participant feedback* Post-program surveys collected quantitative and qualitative feedback on pre-learning modules and workshops, including Likert-scale items (1 = strongly disagree to 5 = strongly agree) assessing satisfaction and perceived impact on practice. Free-text responses captured participants’ reflections on program strengths, areas for improvement, and learning experiences.

*Faculty evolution* We documented faculty composition across years, tracking the evolution from physician-only to multi-professional teaching teams.

*Program organizer reflections* After each workshop, the organizing team conducted structured debriefing sessions capturing implementation challenges, participant responses, and insights for refinement.

*Behavioral change follow-up assessment* For 2023–2024 participants, we conducted 6-month follow-up surveys assessing whether teams established new interprofessional teams for aspiration pneumonia care, including changes that occurred and barriers to implementation (free-text responses).

### Data analysis

*Quantitative data* We calculated descriptive statistics for participant demographics, pre-learning completion rates, and Likert-scale satisfaction scores. We compared completion rates and satisfaction scores across cycles to identify trends.

*Qualitative data* Free-text responses from participant surveys and faculty reflections were analyzed using inductive thematic analysis. Two researchers independently coded the data, identified recurring themes, and resolved discrepancies through discussion. Themes were organized according to Kern’s six-step framework to identify specific areas requiring program modification.

*Integration* Following action research principles, quantitative and qualitative findings were synthesized after each cycle to inform modifications to program design. The research team held reflection meetings to critically examine how findings challenged initial assumptions and what changes were needed for subsequent cycles.

### Ethical considerations

This study received ethical approval from the Institutional Review Board of Hashimoto Municipal Hospital (R2.10-1). All participants provided written informed consent. This study was supported by the GSK Japan Medical Education Grant in 2019. The funding source had no role in study design, data collection, or manuscript preparation. The authors declare no conflicts of interest.

## Results

### Development of the JAPEP workshop: evolution through Kern’s six-step model

Table 2 summarizes how each step of Kern’s model evolved across the three action research phases. The rationale for these modifications is detailed below.

**Table 2:** Evolution of program design through Kern’s 6-step model across action research phases

Phase (year)	Step 1: problem identification	Step 2: targeted needs assessment	Step 3: goals and objectives	Step 4: educational strategies	Step 5: implementation (see Table 3)	Step 6: evaluation
Exploratory (2020)	Low quality of aspiration pneumonia management in older adults due to insufficient physician knowledge	Physicians lack necessary knowledge for management of aspiration pneumonia in older adults	7 domains, 22 competencies (KJ method, physician-focused)	Online webinar (knowledge dissemination)	Individual physicians	Post-webinar surveys
Prototyping (2021)	Despite high prevalence, appropriate interprofessional team collaboration is not occurring	All healthcare professionals lack knowledge beyond their own specialty	7 domains, 23 competencies (reorganized into Knowledge/Skills/Attitude categories; introduced Medication domain, multi-professional focus)	Flipped classroom (individual case scenarios for each module, team-based collaborative learning)	Interprofessional teams from the same hospital	Post-program surveys, pre-learning completion rates, device usage monitoring
Refinement (2022)	Same as prototyping—problem definition stabilized	All healthcare professionals lack knowledge beyond their specialty and lack skills for interprofessional collaboration	12 domains of competency validated by scoping review (99 studies); organized into 8 clinical workflow-aligned modules; enable multi-professional teams to collaboratively apply competencies in patient management	Case-based simulation (virtual interprofessional team managing simulated patient), streamlined pre-learning, mobile-optimized delivery	Interprofessional teams with streamlined allied health pre-learning	Post-program surveys, pre-learning completion rates, 6-month follow-up

### Step 1: problem identification and general needs assessment

During the exploratory phase, we conceptualized the problem as ‘low quality of aspiration pneumonia management in older adults due to insufficient physician knowledge.’ However, pilot webinar data revealed that while physicians recommended the program to other physicians at a mean of 4.38 (1 = strongly disagree to 5 = strongly agree), they more strongly supported multidisciplinary participation with a mean of 4.45. Qualitative feedback included: “There is variation in staff awareness regarding positioning and feeding assistance in aspiration pneumonia care. Quality improvement is necessary,” and “Like physicians, unless the team shares common goals and a certain level of knowledge, it is difficult to provide collaborative care” (Supplementary 1). Therefore, we reframed the problem for the prototyping phase as: ‘Despite high prevalence of aspiration pneumonia patients, appropriate interprofessional team collaboration is not occurring.’ This problem definition remained consistent through the refinement phase, as subsequent evaluation did not reveal need for further modification.

### Step 2: targeted needs assessment

Aligned with the revised problem identification, the targeted needs assessment evolved from ‘physicians lack necessary knowledge for management of aspiration pneumonia in older adults’ in the exploratory phase to all healthcare professionals involved in aspiration pneumonia care lack knowledge beyond their own specialty in the prototyping phase.

During the prototyping phase, participant feedback further revealed deficiencies in collaborative practice skills (Supplementary 2). This highlighted gaps not only in knowledge but also in CoP [24].

In the refinement phase, we therefore expanded the needs assessment to encompass both dimensions: ‘All healthcare professionals involved in aspiration pneumonia management lack knowledge beyond their specialty AND lack skills for interprofessional collaboration.’

### Step 3: goals and objectives

Prior to the exploratory phase, the initial faculty team (2020) used the KJ method—an affinity diagramming technique developed by Japanese anthropologist Jiro Kawakita [24]—to identify 7 domains encompassing 22 competencies for aspiration pneumonia care (Supplementary 4).

However, exploratory phase feedback revealed that these domains lacked external validation and that physicians recommended multidisciplinary staff, rather than physicians alone, should acquire these competencies (Supplementary 1).

Following this feedback, we reorganized the competencies for the prototyping phase, introducing a Medication domain and restructuring content into Knowledge, Skills, and Attitude categories (Supplementary 4). We then conducted a scoping review of 99 studies to develop evidence-based learning domains. This resulted in 12 domains of competency validated by existing literature for the refinement phase: diagnosis, treatment, swallow assessment, underlying condition management, nutrition, oral management, rehabilitation, MDT approach, decision-making, prevention, prognosis, and palliative care. These 12 domains were organized into 8 modules for educational delivery [9].

Prototyping phase feedback and organizer reflections revealed gaps in collaborative practice skills (Supplementary 2). We reframed the goal for the refinement phase to: ‘enabling multiprofessional teams to collaboratively apply the 12 domains of competency in patient care.’

### Step 4: educational strategies

*Exploratory phase (2020)* We implemented an online webinar format for knowledge dissemination to physicians. Eight didactic lectures covered the 22 competencies.

*Prototyping phase (2021)* We adopted a flipped classroom format requiring interprofessional teams from the same hospital to complete pre-learning modules, then participate in facilitated workshops where they practiced collaborative approaches to individual case scenarios for each module.

*Refinement phase (2022)* We implemented enhanced case-based simulation where participants functioned as a virtual interprofessional team caring for a simulated patient across the disease trajectory. However, two implementation challenges emerged: (1) extensive pre-learning created barriers for all participants, particularly allied health professionals with limited professional development time, and (2) prototyping phase data revealed that many participants accessed pre-learning via mobile devices, experiencing connectivity issues. We therefore implemented three key modifications: streamlined pre-learning with essential content for interprofessional collaboration, using the same modules for all participants but focusing on core knowledge necessary for team-based approach; mobile-optimized delivery, including reduced video quality for better streaming and a mobile-friendly Moodle learning management system theme to improve accessibility; and a weekly reminder system with automated emails containing direct access links to address completion challenges.

### Step 5: implementation

Table 3 summarizes the educational programs implemented across phases. From the prototyping phase onward, we required participation as interprofessional teams from the same hospital to facilitate post-workshop implementation. The teaching faculty evolved from physician-only to multi-professional across the study period. Faculty members were selected from healthcare professionals with clinical or research experience in aspiration pneumonia care at their institutions. Candidates expressed interest in joining the faculty following presentations of the program at academic and clinical meetings, and final selection was made by the program leaders based on their expertise and institutional engagement.

**Table 3:** Educational program structure and faculty composition across action research phases

Phase (year)	Format	Duration	Pre-learning	Participant structure	Workshop activity	Faculty (total, disciplines)
Exploratory (2020)	Online webinar	4 h	None	Individual physicians	Didactic lectures	9 MDs, 1 MD/DDS (*n* = 10, 2 disciplines)
Prototyping (2021)	Flipped classroom	Pre-learning + 8-h workshop	Comprehensive modules for all	Hospital-based interprofessional teams	Individual case scenarios for each module	9 MDs, 1 MD/DDS, 1 Ph (*n* = 11, 3 disciplines)
Refinement (2022)	Flipped classroom with simulation	Pre-learning + 8-h workshop	Streamlined essential content for all participants (focused on interprofessional collaboration); mobile-optimized delivery	Hospital-based interprofessional teams	Case-based team simulation	8 MDs, 1 MD/DDS, 1 Ph, 1 RD (*n* = 11, 4 disciplines)
Implementation (2023—2024)	Flipped classroom with simulation	Pre-learning + 8-h workshop	Streamlined essential content for all participants (focused on interprofessional collaboration); mobile-optimized delivery	Hospital-based interprofessional teams	Case-based team simulation	7 MDs, 1 MD/DDS, 1 Ph, 1 RD, 2 DDS, 1 NP, 1 ST (*n* = 14, 6 disciplines)

DDS, dentist; MD, physician; NP, nurse practitioner; PharmD, pharmacist; RD, dietitian; RN, nurse; SLT, speech and language therapist

### Step 6: evaluation and feedback

At each phase, we collected both quantitative and qualitative data to assess learning outcomes and program implementation. Following implementation of the flipped classroom format, we monitored pre-learning completion rates in addition to post-program evaluation surveys.

Overall satisfaction scores were 4.4 ± 0.6 (response rate 82.9%, 29/35) in the exploratory phase, 4.5 ± 0.5 (response rate 84.4%, 38/45) in the prototyping phase, and 4.5 ± 0.6 (response rate 84.3%, 43/51) in the refinement phase. Detailed satisfaction data and pre-learning completion rates across all phases are available in Supplementary materials 1, 2, and 3.

Pre-learning completion rates improved substantially across phases: 84.4% (38/45) in the prototyping phase, 96.1% (49/51) in the refinement phase, and 96.8% (122/126) in the implementation phase. This improvement was attributed to the weekly reminder system and mobile optimization introduced in the refinement phase.

Key themes from qualitative feedback at each phase informed subsequent modifications (see Supplementary 2 and 3).

### Final structure of the JAPEP workshop

**Fig. 1:**
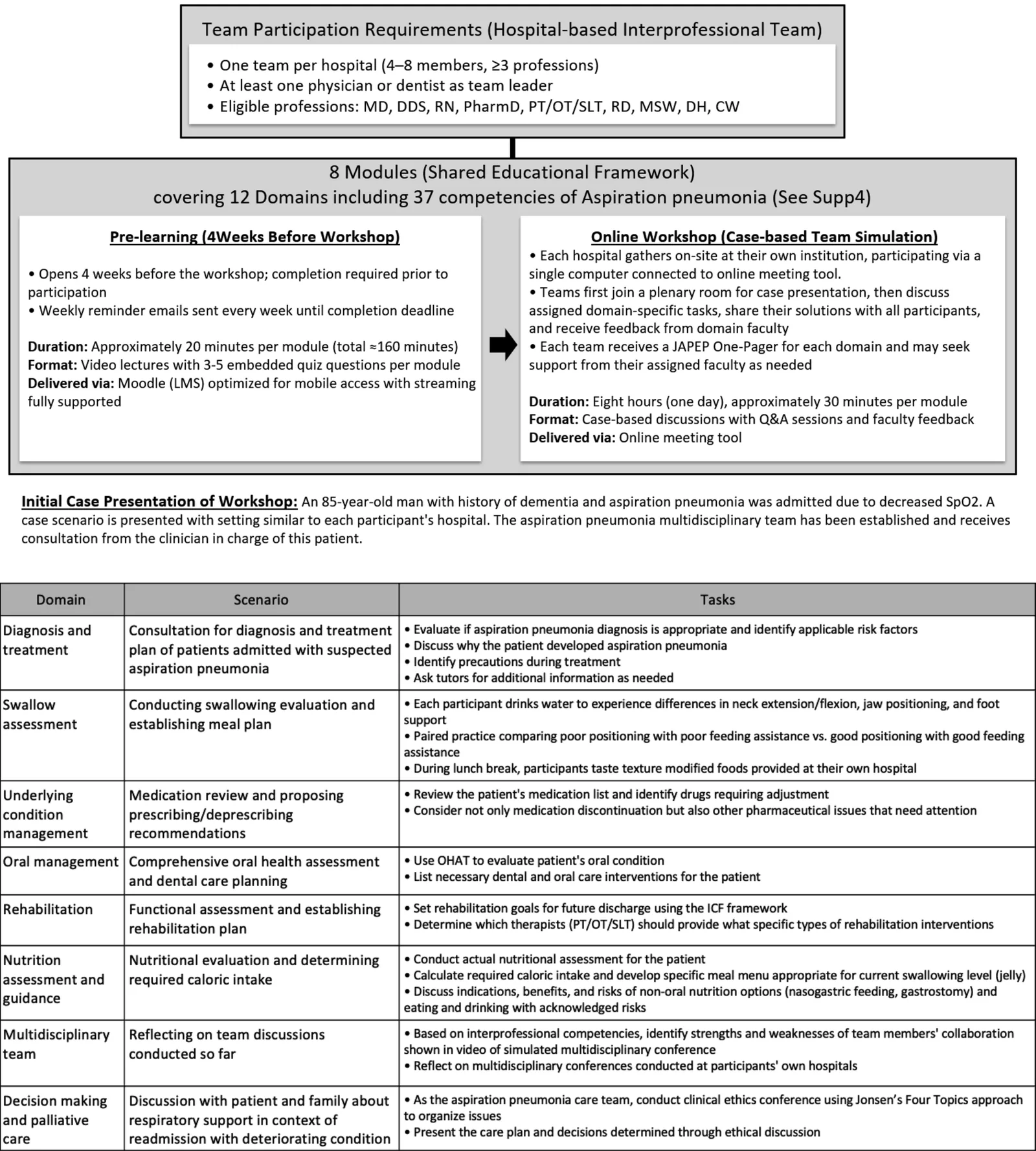
Structure of the JAPEP multi-professional educational program. CW, care worker; DDS, dentist; DH, dental hygienist; ICF, International Classification of Functioning, Disability and Health [25]; JAPEP, Japan Aspiration pneumonia inter-Professional team Educational Program; Johnson’s Four Topics approach [26]; LMS, learning management system; MD, physician; MSW, medical social worker; OHAT, Oral Health Assessment Tool [27]; Ph, pharmacist; PT/OT/SLT, physical/occupational/speech and language therapist; RD, registered dietitian; RN, registered nurse

Through three action research phases, we developed a finalized multi-professional educational program. The program structure is illustrated in Fig. 1. The final program consisted of streamlined pre-learning modules (essential content focused on interprofessional collaboration which was provided to all participants) followed by team-based workshops utilizing case-based simulation, facilitated by a multidisciplinary faculty team.

### Follow-up assessment of practice implementation

During the program development phase (2020–2022), we only evaluated participant satisfaction (Kirkpatrick Level 1) [28]. To assess whether the educational intervention led to behavioral changes in clinical practice, we conducted a 6-month follow-up of teams participating in the implementation stage (2023–2024), corresponding to Kirkpatrick Level 3 evaluation [29]. We specifically assessed whether teams established in-hospital MDTs for aspiration pneumonia management.

Of the 15 participating teams, 10 responded (67% response rate). Among eight teams without pre-existing aspiration pneumonia MDTs, three (37.5%) established new teams within 6 months. The two teams with existing MDTs reported enhanced activities. Five teams cited barriers to MDT formation, with time constraints being the most prevalent (80%). Results are summarized in Table 4.

**Table 4:** Six-month follow-up survey results (*n* = 10 responding teams)

Pre-existing MDT status	Outcome	*n*	Details
No pre-existing MDT	New MDT established	3/8	Team composition: physicians, dysphagia-certified nurses, dietitians (all teams); ± physical therapist, speech and language therapists, pharmacists, dentists. Activities: multidisciplinary meetings, team rounds, swallow assessments, staff education
	MDT not established	5/8	Barriers: time constraints (*n* = 2), unclear roles/responsibilities (*n* = 2), low meeting attendance (*n* = 2), communication gaps (*n* = 1), unclear decision-making (*n* = 1), lack of trust (*n* = 1), hierarchical relationships (*n* = 1)
Pre-existing MDT	Existing MDT enhanced	2/2	Changes: increased junior staff participation, enhanced oral hygiene focus, standardized care protocols

Survey context: response rate 10/15 teams (67%); teams without pre-existing MDT: 8/10 (80%); teams with pre-existing MDT: 2/10 (20%)

*MDT* multidisciplinary team

All newly formed teams adopted consistent core membership comprising a physician, a dysphagia-certified nurse, and a dietitian, with foundational activities including regular multidisciplinary meetings and team rounds. Among teams unable to establish formal MDTs, behavioral changes were nonetheless evident, including enhanced assessment skills, improved communication, and ongoing informal interprofessional collaboration.

### Reflections on the action research process

The central reflections emerging from this evolutionary process are as follows:

### Paradigm shift from knowledge deficit to collaborative practice

Our action research revealed a fundamental misconception in our initial framing. We initially assumed knowledge deficits were the primary barrier. However, feedback shifted our understanding toward a sociocultural learning paradigm, recognizing that effective management requires functioning as a CoP [20].

This represents transformative learning [22]—rather than viewing quality as a technical problem requiring knowledge transfer, we understood it as an adaptive challenge requiring interprofessional collaboration. This reframing had profound implications for all subsequent steps in Kern’s model.

### Importance of situated learning

The evolution from webinar to flipped classroom to case-based simulation reflects increasing recognition of Situated Learning Theory [21]. Our developmental cycle revealed that learning interprofessional collaboration cannot occur in abstract, decontextualized settings. By bringing intact teams from the same hospitals and having them work through realistic patient scenarios together, we created conditions for legitimate peripheral participation—allowing participants to engage in authentic collaborative practice within a supportive learning environment.

The case-based simulation exemplifies this principle: participants experienced collaborative decision-making, negotiated professional boundaries, and developed shared mental models while caring for a simulated patient—activities directly transferable to clinical practice.

### Balancing comprehensiveness with accessibility

While comprehensive pre-learning ensured foundational knowledge, completion data revealed it created barriers for allied health professionals with limited professional development time. Our solution—streamlined pre-learning with essential content—acknowledges that effective collaboration requires complementary expertise with sufficient overlap for mutual understanding, aligning with the concept of boundary objects [30].

### Device accessibility and blended learning design

Analysis of pre-learning completion patterns revealed that a substantial proportion of participants accessed materials via mobile devices (50.0% in 2021, 58.2% in 2022), with smartphones being primary (44.7% in 2021, 53.5% in 2022). The prototyping phase identified significant mobile accessibility barriers. By implementing mobile-optimized content delivery in the refinement phase, pre-learning completion rates improved from 84.4 to 96.1%. This finding underscores that blended learning programs must be designed with explicit consideration of learners’ device preferences [31, 32].

## Discussion

We conducted an action research study to develop an educational program training multidisciplinary healthcare professionals in comprehensive management of aspiration pneumonia in older adults. The primary aim was achieved through participant satisfaction, MDT development, and reported behavioral changes. Critical reflections on qualitative and quantitative feedback through the action research model enabled development of an educational curriculum promoting MDT formation specialized in aspiration pneumonia management. As previously reported, medical education has predominantly focused on curative medicine, with huge gaps in areas such as multidisciplinary team work, end-of-life care, and shared decision-making [8].

The essential elements of a satisfactory and efficient online MDT training program included MDT participation from the same setting, pre-course learning to level the basic understanding among disciplines and varying degrees of experience, accessibility of pre-course materials, and mobile optimization and device accessibility to accommodate learners’ actual access patterns. It is also noteworthy that critical analysis from structured feedback is an invaluable form of reflecting on the program and refining the focus and design.

Another important objective was to explore interventions facilitating practice change, namely MDT formation. The need for MDT education and simulation training has been reported in various studies, including studies in eating and drinking with acknowledged risks in older adults [8, 33]. The most significant finding was the demand for a paradigm shift in educational focus from knowledge deficit to collaborative practice. Reasons participants raised for forming or not forming MDTs in the follow-up survey (Table 4) reveal the importance of shared understanding and mission within institutions, aligning with CoP principles [20]. We developed our training program emphasizing MDT training from a CoP perspective to build shared understanding and teamwork. Particularly important were voluntary participation based on shared interest, organizational knowledge, and communication and management, which align with critical factors of CoP development [34] and are transferable to MDT training in other healthcare areas. Reported barriers towards MDT formation—including lack of resources, lack of shared understanding, and differing motivation—can mostly be attributed to lack of CoP. Nurturing CoP in each setting will be a major future challenge.

To our knowledge, no studies provide definitive evidence of a sustained, member-led, non-virtual hospital team CoP-initiated by a formal curriculum. Common pitfalls with CoP, including the risk of conflation of educational programs and CoP, insufficient outcomes, and dependence on facilitator-led curricula must be addressed when developing programs, to facilitate sustainable practice change.

This study has important implications in clinical and academic fields.

In an aging society where the largest patient population is over 65, managing older adults with aspiration pneumonia is crucial medically, socially, and economically. Aspiration pneumonia is a complex syndrome requiring active MDT involvement throughout the disease course, with unique training needs for each team member [8, 9]. Traditional, passive educational methods are less effective in nurturing skills needed to approach these difficult cases comprehensively. New methods, including active MDT participation combining online learning, simulation training, and case-based discussions to build CoP, effectively address these challenges.

Effective evidence-based MDT training will hopefully facilitate appropriate acknowledgement and reimbursement. Japan’s Ministry of Health, Labour, and Welfare has introduced initiatives such as additional insurance points for MDT-based dysphagia care. However, conditions remain stringent. These structural barriers have been compounded by chronic workforce shortages, critically low preventive dental care utilization among older adults (18.6%), and aspiration pneumonia accounting for approximately 70% of pneumonia cases in Japanese nursing facilities [35, 36]. Many clinical settings cannot utilize this system. With more MDTs trained through our program, we expect increased ability to comply with these restrictions, enabling proper reimbursement for quality care. Furthermore, we anticipate addressing shortcomings of add-on fees and expanding scope to include complex situations such as shared decision-making, care transitions, and end-of-life care, contributing to a healthcare system supporting the complex needs of a super-aged society.

Some limitations must be mentioned, including the relatively small sample size and short follow-up period, and selection bias potentially limiting generalizability. However, there were voluntary participants from hospitals across Japan, with good response rates. Culturally and socially sensitive adaptations depending on setting or country may also be necessary. Additionally, this study was conducted exclusively in hospital settings; training objectives, skills, and competencies may differ in community, primary care, and long-term care environments, and future research should explore the adaptation of this program to such settings.

The program evaluated participant satisfaction (Kirkpatrick Level 1) and behavioral change (Kirkpatrick Level 3) but did not include formal summative learner assessment to verify competency attainment. Future iterations should incorporate structured assessment tools, such as simulation-based competency evaluation, to confirm that learning objectives have been met. Furthermore, patient-level clinical outcomes, including incidence of aspiration pneumonia, in-hospital mortality, and morbidity, were not measured following MDT formation. Larger prospective studies incorporating such outcome data are warranted to establish the clinical effectiveness of this educational intervention.

## Conclusion

We developed an educational intervention to train MDTs in the management of aspiration pneumonia in older adults. The extensive action research process enabled identification of key components towards an effective educational intervention. This training program will be invaluable in supporting the increasingly complex training needs of healthcare professionals facing the challenges of an aging society.

## Supplementary Information

Below is the link to the electronic supplementary material.

Supplementary file1 (DOCX 78 KB)

## Data Availability

The datasets generated and/or analyzed during the current study are available from the corresponding author on reasonable request.
