# Supplementary material for: Development of an interprofessional education program for aspiration pneumonia care: an action research study
Source: Eur Geriatr Med. 2026 Aug 10;17(5):2907–18. doi: 10.1007/s41999-026-01490-2 (PMC13754991; doi:10.1007/s41999-026-01490-2)
Supplement: Supplementary file 1 [file 41999_2026_1490_MOESM1_ESM.docx]

# Supplementary 1: Exploratory Phase (2020) - Post-Webinar Evaluation Results

## Participant Characteristics and Educational Intervention

| Characteristic | Details |
| --- | --- |
| Total participants | 35 physicians |
| Format | Online webinar |
| Duration | Approximately 4 hours |
| Content | Seven topics covering aspiration pneumonia management: Diagnosis and Treatment, Swallow Assessment, Rehabilitation, Nutrition, Oral/Dental Care, Ethical Issues, Multidisciplinary Collaboration |

## Survey Methods

Data collection: Google Forms, Data analysis: Microsoft Excel

## Response Rate

Post-webinar survey response rate: 29/35 (82.9%)

## Survey Respondent Characteristics

All participants in this webinar were physicians; however, personal information such as years of clinical experience was not collected.

## Quantitative and Qualitative Data

### Overall

#### Overall Satisfaction

| Response | n | % |
| --- | --- | --- |
| Very satisfied (5) | 14 | 48.3 |
| Satisfied (4) | 13 | 44.8 |
| Neither satisfied nor dissatisfied (3) | 2 | 6.9 |
| Dissatisfied (2) | 0 | 0.0 |
| Very dissatisfied (1) | 0 | 0.0 |

Mean ± SD: 4.41 ± 0.62 / 5.0

#### Recommendation to Other Physicians

| Response | n | % |
| --- | --- | --- |
| Strongly agree (5) | 15 | 51.7 |
| Agree (4) | 10 | 34.5 |
| Neither agree nor disagree (3) | 4 | 13.8 |
| Disagree (2) | 0 | 0.0 |
| Strongly disagree (1) | 0 | 0.0 |

Mean ± SD: 4.38 ± 0.72 / 5.0

#### Recommendation to Other Healthcare Professionals

| Response | n | % |
| --- | --- | --- |
| Strongly agree (5) | 15 | 51.7 |
| Agree (4) | 12 | 41.4 |
| Neither agree nor disagree (3) | 2 | 6.9 |
| Disagree (2) | 0 | 0.0 |
| Strongly disagree (1) | 0 | 0.0 |

Mean ± SD: 4.45 ± 0.62 / 5.0

#### Free Comments on the Webinar

Theme 1: Need for Common Language and Shared Understanding

Establishing a common language and shared understanding is essential for effective interprofessional collaboration in aspiration pneumonia prevention.

"If we can establish a common language and shared understanding within the hospital, interprofessional collaboration becomes much easier."

"With this foundational knowledge, we can have deeper discussions in case conferences because we share a common language."

Theme 2: Understanding Professional Roles

Understanding each profession's specific roles and sharing this knowledge across disciplines enhances healthcare workers' willingness to participate in interprofessional collaboration.

"In dysphagia care, it would be beneficial for each profession to understand their own roles."

"If lectures on profession-specific skills were delivered by those professionals themselves, other healthcare workers might be more interested in participating."

Theme 3: Team-Based Approach Requires Shared Knowledge

Team-based collaborative approach requires all healthcare professions to share common goals and a certain level of knowledge.

"Like physicians, unless the team shares common goals and a certain level of knowledge, it is difficult to provide collaborative care."

"I believe aspiration pneumonia management requires awareness and knowledge across a wide range of healthcare professions."

Theme 4: Variation in Staff Awareness and Skills

Variations exist in staff awareness and skills regarding aspiration pneumonia management across different professions and individuals, necessitating quality improvement.

"There is variation in staff awareness regarding positioning and feeding assistance in aspiration pneumonia care. Quality improvement is necessary."

## Key Findings

1. High satisfaction: 93.1% of participants were satisfied (44.8%) or very satisfied (48.3%).
2. Strong recommendation for multiprofessional participation
3. Recognition of team-based care needs: Qualitative feedback consistently emphasized:
4. Paradigm shift identified: These findings prompted the research team to reframe the problem from ’physician knowledge deficit‘ to ’need for interprofessional team collaboration.’

## Implications for Program Development

Based on these findings, the program evolved from:

- Target audience: Physicians only → Multidisciplinary teams
- Problem definition: Low quality due to insufficient physician knowledge → Lack of appropriate interprofessional team collaboration
- Educational approach: Individual learning → Team-based learning with members from the same hospital

This feedback was the primary evidence supporting the paradigm shift described in Step 1 (Problem Identification and General Needs Assessment) of the main manuscript.

# Supplementary 2: Prototyping Phase (2021) - Post-Program Evaluation Results

## Participant Characteristics and Educational Intervention

| Characteristic | Details |
| --- | --- |
| Total participants | 45 participants from 6 hospitals |
| Team composition | Hospital-based interprofessional teams |
| Format | Flipped classroom (streamlined pre-learning + online workshop) |
| Duration | 8 hours workshop + pre-learning modules |

## Survey Methods

Data collection: Google Forms, Data analysis: Microsoft Excel

## Response Rate

Post-program survey response rate: 38/45 (84.4%)

## Survey Respondent Characteristics

Note: The following data represents survey respondents (n=38), not all participants (n=45). Response rate: 84.4%

### Professional Role

| Role | n | % |
| --- | --- | --- |
| Nurse | 8 | 21.1 |
| Speech and Language Therapist | 7 | 18.4 |
| Physical Therapist | 6 | 15.8 |
| Physician | 4 | 10.5 |
| Pharmacist | 4 | 10.5 |
| Dietitian | 3 | 7.9 |
| Occupational Therapist | 3 | 7.9 |
| Dentist | 2 | 5.3 |
| Social Worker | 1 | 2.6 |

### Years of Clinical Experience

| Experience | n | % |
| --- | --- | --- |
| 1-2 years | 3 | 7.9 |
| 3 years | 1 | 2.6 |
| 4 years | 2 | 5.3 |
| 5 years | 1 | 2.6 |
| 6-9 years | 3 | 7.9 |
| 10-19 years | 17 | 44.7 |
| ≥20 years | 11 | 28.9 |

## Quantitative and Qualitative Data

### Overall

#### Overall Satisfaction

| Response | n | % |
| --- | --- | --- |
| Very satisfied (5) | 17 | 44.7 |
| Satisfied (4) | 21 | 55.3 |
| Neither satisfied nor dissatisfied (3) | 0 | 0.0 |
| Dissatisfied (2) | 0 | 0.0 |
| Very dissatisfied (1) | 0 | 0.0 |

Mean ± SD: 4.45 ± 0.50 / 5.0

#### Recommendation to Other Professionals in Same Discipline

| Response | n | % |
| --- | --- | --- |
| Strongly agree (5) | 18 | 47.4 |
| Agree (4) | 20 | 52.6 |
| Neither agree nor disagree (3) | 0 | 0.0 |
| Disagree (2) | 0 | 0.0 |
| Strongly disagree (1) | 0 | 0.0 |

Mean ± SD: 4.47 ± 0.50 / 5.0

#### Recommendation to Other Physicians and Dentists

| Response | n | % |
| --- | --- | --- |
| Strongly agree (5) | 19 | 50.0 |
| Agree (4) | 16 | 42.1 |
| Neither agree nor disagree (3) | 3 | 7.9 |
| Disagree (2) | 0 | 0.0 |
| Strongly disagree (1) | 0 | 0.0 |

Mean ± SD: 4.42 ± 0.63 / 5.0

### Pre-learning

Pre-learning completion rate: 38/45 (84.4%)

#### Time Required to Complete Pre-learning

| Time required | n | % |
| --- | --- | --- |
| Within 1 week | 5 | 13.2 |
| 1-2 weeks | 7 | 18.4 |
| 2-4 weeks | 20 | 52.6 |
| More than 4 weeks | 6 | 15.8 |

#### Perceived Difficulty

| Response | n | % |
| --- | --- | --- |
| Very difficult (1) | 0 | 0.0 |
| Difficult (2) | 6 | 15.8 |
| Appropriate (3) | 32 | 84.2 |
| Easy (4) | 0 | 0.0 |
| Very easy (5) | 0 | 0.0 |

Mean ± SD: 2.84 ± 0.36 / 5.0

#### Perceived Volume

| Response | n | % |
| --- | --- | --- |
| Far too much (5) | 3 | 7.9 |
| Too much (4) | 20 | 52.6 |
| Appropriate (3) | 15 | 39.5 |
| Too little (2) | 0 | 0.0 |
| Far too little (1) | 0 | 0.0 |

Mean ± SD: 3.68 ± 0.61 / 5.0

#### Device Usage for Pre-learning

| Device type | n | % |
| --- | --- | --- |
| Smartphone | 17 | 44.7 |
| Tablet | 2 | 5.3 |
| Desktop/Laptop computer | 19 | 50.0 |

#### Free Comments on Pre-learning

Theme 1: Time Burden

The comprehensive pre-learning content required significant time commitment that was challenging to manage alongside busy clinical work schedules.

"The pre-learning content was comprehensive but required significant time commitment during busy clinical work."

"As a nurse, I found it challenging to dedicate 2-4 weeks to complete all modules while managing shift work."

Theme 2: Mobile Access Challenges

Technical difficulties with mobile device access, including streaming interruptions and platform optimization issues, hindered completion of pre-learning modules.

"I wanted to watch videos during my commute, but the streaming was often interrupted."

"The platform was not optimized for smartphone viewing, making it difficult to complete modules on mobile devices."

Theme 3: Completion Reminders Needed

Busy clinicians sometimes forgot to complete pre-learning modules without systematic reminders.

"As busy clinicians, we sometimes forgot to complete the pre-learning modules without reminders."

### Workshop

#### Satisfaction with Live Workshop

| Response | n | % |
| --- | --- | --- |
| Very satisfied (5) | 17 | 44.7 |
| Satisfied (4) | 20 | 52.6 |
| Neither satisfied nor dissatisfied (3) | 1 | 2.6 |
| Dissatisfied (2) | 0 | 0.0 |
| Very dissatisfied (1) | 0 | 0.0 |

Mean ± SD: 4.42 ± 0.54 / 5.0

#### Perceived Workshop Duration

| Response | n | % |
| --- | --- | --- |
| Far too long | 0 | 0.0 |
| Too long | 3 | 7.9 |
| Appropriate | 32 | 84.2 |
| Too short | 3 | 7.9 |
| Far too short | 0 | 0.0 |

#### Free Comments on Workshop

Theme 1: Value of Interprofessional Discussion

Interprofessional discussions revealed the difficulty of understanding different professional perspectives and the lack of common language across disciplines in patient management.

"I found it difficult to understand the perspectives and priorities of other professions during case discussions."

"We lacked a common language when discussing patient care across disciplines."

Theme 2: Need for Practical Collaborative Skills

Participants identified challenges in practical collaboration, including unclear role boundaries, leadership ambiguity, and the complexity of integrating diverse professional opinions into unified care plans.

"It was sometimes unclear which profession should take the lead on specific aspects of care."

"Role boundaries were not always clear when multiple professions could contribute to the same aspect of care."

"We struggled with how to integrate different professional opinions into a unified care plan."

"Decision-making as a team was more challenging than I anticipated."

## Organizer's Reflection

After analyzing the 2021 prototyping phase evaluation data and qualitative feedback, the organizing team identified several key insights:

### Successful Elements to Maintain

1. Multiprofessional team format: The shift from physician-only (2020) to interprofessional teams was validated by 100% satisfaction rate and consistent high scores across all recommendation metrics.

2. Flipped classroom approach: The combination of pre-learning and interactive workshop was well-received, though implementation challenges emerged.

### Critical Issues Requiring Intervention

1. Pre-learning time burden:

- 52.6% required 2-4 weeks to complete
- 60.5% perceived volume as excessive
- Particularly problematic for allied health professionals with limited professional development time
- Proposed solution: Differentiate pre-learning content by professional role

2. Mobile access limitations:

- 50% accessed via mobile devices but experienced technical difficulties
- Platform not optimized for smartphone viewing
- Proposed solution: Mobile-optimized content delivery

3. Completion rate challenges:

- Busy clinicians reported forgetting to complete modules
- No systematic reminder system in place
- Proposed solution: Implement weekly reminder emails with direct access links

4. Collaborative practice skills gap:

- Participants recognized gaps not only in knowledge but also in skills for interprofessional collaboration
- Workshop format needed enhancement to practice team-based decision-making
- Proposed solution: Introduce case-based simulation focused on collaborative skills

## Key Findings

### 1. Validation of Multiprofessional Team Format

The shift from physician-only to multiprofessional team format was successful. The paradigm shift from ’physician knowledge deficit‘ to ’need for interprofessional collaboration‘ was validated.

### 2. Pre-learning Implementation Challenges

While pre-learning content quality was appropriate, implementation barriers related to volume, mobile accessibility, and completion support needed to be addressed in the refinement phase.

### 3. Recognition of Collaborative Practice Skills Gap

This finding led to expanded needs assessment and informed the decision to implement case-based simulation in the refinement phase to provide practice in collaborative skills, not just knowledge transfer.

## Implications for Refinement Phase (2022)

Based on prototyping phase findings, the following modifications were planned for implementation:

1. Streamlined pre-learning: Streamline content for allied health professionals to reduce time burden while maintaining comprehensive modules for physicians
2. Mobile optimization: Redesign learning platform for optimal mobile device access and video streaming
3. Weekly reminder system: Implement automated weekly reminder emails with direct access links to pre-learning modules
4. Case-based simulation: Enhance workshop with structured case simulation focused on practicing collaborative skills and team decision-making
5. Multiprofessional faculty expansion: Increase representation of non-physician faculty to model interprofessional collaboration throughout the program

# Supplementary 3: Refinement Phase (2022) - Post-Program Evaluation Results

## Participant Characteristics and Educational Intervention

| Characteristic | Details |
| --- | --- |
| Total participants | 51 participants from 8 hospitals |
| Team composition | Hospital-based interprofessional teams |
| Format | Flipped classroom with case-based simulation (streamlined pre-learning + online workshop) |
| Duration | 8 hours workshop + pre-learning modules |

## Survey Methods

Data collection: Google Forms, Data analysis: Microsoft Excel

## Response Rate

Post-program survey response rate: 43/51 (84.3%)

## Survey Respondent Characteristics

Note: The following data represents survey respondents (n=43), not all participants (n=51). Response rate: 84.3%

### Professional Role

| Role | n | % |
| --- | --- | --- |
| Physician | 8 | 18.6 |
| Nurse | 7 | 16.3 |
| Dietitian | 7 | 16.3 |
| Speech and Language Therapist | 6 | 14.0 |
| Physical Therapist | 6 | 14.0 |
| Pharmacist | 4 | 9.3 |
| Dental Hygienist | 1 | 2.3 |
| Social Worker | 1 | 2.3 |
| Nurse Practitioner | 1 | 2.3 |
| Dentist | 1 | 2.3 |
| Occupational Therapist | 1 | 2.3 |

### Years of Clinical Experience

| Experience | n | % |
| --- | --- | --- |
| 1-2 years | 3 | 7.0 |
| 3-5 years | 11 | 25.6 |
| 6-9 years | 8 | 18.6 |
| 10-19 years | 13 | 30.2 |
| ≥20 years | 8 | 18.6 |

## Quantitative and Qualitative Data

### Overall

#### Overall Satisfaction

| Response | n | % |
| --- | --- | --- |
| Very satisfied (5) | 22 | 51.2 |
| Satisfied (4) | 20 | 46.5 |
| Neither satisfied nor dissatisfied (3) | 1 | 2.3 |
| Dissatisfied (2) | 0 | 0.0 |
| Very dissatisfied (1) | 0 | 0.0 |

Mean ± SD: 4.49 ± 0.55 / 5.0

#### Recommendation to Other Professionals in Same Discipline

| Response | n | % |
| --- | --- | --- |
| Strongly agree (5) | 20 | 50.0 |
| Agree (4) | 17 | 42.5 |
| Neither agree nor disagree (3) | 2 | 5.0 |
| Disagree (2) | 1 | 2.5 |
| Strongly disagree (1) | 0 | 0.0 |

Mean ± SD: 4.40 ± 0.70 / 5.0 (n=40; 3 participants responded "Not applicable")

#### Recommendation to Other Physicians and Dentists

| Response | n | % |
| --- | --- | --- |
| Strongly agree (5) | 24 | 60.0 |
| Agree (4) | 15 | 37.5 |
| Neither agree nor disagree (3) | 0 | 0.0 |
| Disagree (2) | 1 | 2.5 |
| Strongly disagree (1) | 0 | 0.0 |

Mean ± SD: 4.55 ± 0.63 / 5.0 (n=40; 3 participants responded "Not applicable")

### Pre-learning

Pre-learning completion rate: 49/51 (96.1%)

#### Time Required to Complete Pre-learning

| Time required | n | % |
| --- | --- | --- |
| Within 1 week | 11 | 27.5 |
| 1-2 weeks | 8 | 20.0 |
| 2-4 weeks | 16 | 40.0 |
| More than 4 weeks | 5 | 12.5 |

(n=40; 3 participants responded "Not applicable")

#### Perceived Difficulty

| Response | N | % |
| --- | --- | --- |
| Very difficult (1) | 0 | 0.0 |
| Difficult (2) | 8 | 20.0 |
| Appropriate (3) | 32 | 80.0 |
| Easy (4) | 0 | 0.0 |
| Very easy (5) | 0 | 0.0 |

Mean ± SD: 2.80 ± 0.40 / 5.0 (n=40; 3 participants responded "Not applicable")

#### Perceived Volume

| Response | n | % |
| --- | --- | --- |
| Far too much (5) | 1 | 2.5 |
| Too much (4) | 12 | 30.0 |
| Appropriate (3) | 26 | 65.0 |
| Too little (2) | 1 | 2.5 |
| Far too little (1) | 0 | 0.0 |

Mean ± SD: 3.33 ± 0.57 / 5.0 (n=40; 3 participants responded "Not applicable")

#### Perceived Effect of Weekly Reminder System

| Response | n | % |
| --- | --- | --- |
| Strongly agree (5) | 7 | 17.5 |
| Agree (4) | 31 | 77.5 |
| Neither agree nor disagree (3) | 1 | 2.5 |
| Disagree (2) | 1 | 2.5 |
| Strongly disagree (1) | 0 | 0.0 |

Mean ± SD: 4.10 ± 0.54 / 5.0 (n=40; 3 participants responded "Not applicable")

#### Device Usage for Pre-learning

| Device type | n | % |
| --- | --- | --- |
| Smartphone | 23 | 53.5 |
| Tablet | 2 | 4.7 |
| Desktop/Laptop computer | 18 | 41.9 |

#### Free Comments on Pre-learning

Theme 1: Effectiveness of Streamlined Content

Streamlined pre-learning content allowed participants to focus on role-specific information while maintaining interprofessional awareness.

"As a nurse, the streamlined pre-learning content was much more manageable than I expected. It covered what I needed without overwhelming detail."

" I could focus on content relevant to my role while still understanding the broader picture."

Theme 2: Mobile Optimization Success

Mobile optimization significantly improved accessibility, enabling participants to complete modules conveniently during commute time with smooth video streaming and easy navigation.

"Being able to complete modules on my smartphone during commute time was incredibly convenient."

"The mobile optimization made a huge difference - videos streamed smoothly and the interface was easy to navigate on my phone."

Theme 3: Value of Reminder System

Weekly reminders with direct links helped busy clinicians stay on track and complete pre-learning modules systematically despite their demanding clinical schedules.

"The weekly reminders with direct links kept me on track. Without them, I might have forgotten in the midst of busy clinical work."

"Having reminders helped me plan my time better and complete the modules systematically."

### Workshop

#### Satisfaction with Live Workshop

| Response | n | % |
| --- | --- | --- |
| Strongly agree (5) | 15 | 37.5 |
| Agree (4) | 24 | 60.0 |
| Neither agree nor disagree (3) | 1 | 2.5 |
| Disagree (2) | 0 | 0.0 |
| Strongly disagree (1) | 0 | 0.0 |

Mean ± SD: 4.35 ± 0.53 / 5.0 (n=40; 3 participant responded "Not applicable")

#### Perceived Workshop Duration

| Response | n | % |
| --- | --- | --- |
| Far too long | 1 | 2.5 |
| Too long | 8 | 20.0 |
| Appropriate | 29 | 72.5 |
| Too short | 1 | 2.5 |
| Far too short | 1 | 2.5 |

(n=40; 3 participant responded "Not applicable")

#### Free Comments on Workshop

Theme 1: Value of Case-Based Simulation

Case-based simulation enabled practical team collaboration through realistic scenarios with colleagues from different professions.

"The case simulation allowed us to practice working together as a team, not just learning about it theoretically."

"I appreciated the opportunity to work through realistic scenarios with colleagues from different professions."

Theme 2: Enhanced Collaborative Skills

Structured simulation clarified professional roles and taught practical strategies for integrating diverse perspectives into unified care plans.

"The structured simulation helped clarify our roles and how to coordinate care effectively."

"I learned practical strategies for integrating different professional perspectives into a unified care plan."

Theme 3: Interprofessional Learning

Observing how other professions approach patient care provided unique insights unattainable in single-profession settings.

"Hearing how other professions approach the same patient taught me things I never would have learned in a single-profession setting."

"The multiprofessional faculty modeling collaboration throughout the workshop was powerful."

## Organizer's Reflection

After implementing the 2022 refinement phase modifications, the organizing team reflected on the outcomes:

### Successful Interventions Validated by Data

1. Streamlined pre-learning:

- Volume perception improved dramatically from 60.5% (2021) perceiving excessive volume to only 32.5% (2022)
- Time to completion decreased: 52.6% requiring 2-4 weeks (2021) vs 40.0% (2022)
- Completion rate within 2 weeks improved from 31.6% (2021) to 47.5% (2022; 27.5% within 1 week, 20.0% in 1-2 weeks)
- Outcome: Content streamlining successfully reduced burden without compromising quality

2. Weekly reminder system:

- 95.0% found reminders effective (Mean: 4.10 ± 0.54)
- Pre-learning completion rate improved from 84.4% (2021) to 96.1% (2022)
- Addressed the ’busy clinician forgetting‘ issue identified in 2021
- Outcome: Simple, low-cost intervention with high participant endorsement and measurable impact on completion

3. Mobile optimization:

- Mobile device usage increased from 50.0% (2021) to 58.2% (2022)
- Smartphone usage specifically increased from 44.7% to 53.5%
- Qualitative feedback confirmed improved mobile experience
- Outcome: Technical improvements successfully enhanced accessibility

4. Case-based simulation:

- Qualitative feedback emphasized value of practicing collaborative skills
- Participants reported practical strategies for team coordination
- Outcome: Successfully addressed the collaborative practice skills gap identified in 2021

### Remaining Opportunities for Improvement

1. Platform satisfaction:

- While functional (4.07 ± 0.81), platform satisfaction was lower than content satisfaction (4.35 for lectures)
- 16.7% neutral, 4.8% disagree suggests room for further enhancement
- Future consideration: Platform user experience refinement

2. Pre-learning completion time:

- While improved, 40.0% still required 2-4 weeks
- 12.5% required more than 4 weeks
- Future consideration: Further streamlining or extended pre-program timeline

3. Workshop duration perception:

- Despite identical 8-hour duration as 2021, more participants perceived the workshop as "too long" (7.9% in 2021 vs 22.5% in 2022)
- This likely reflects increased cognitive load from the enhanced case-based simulation requiring deeper collaborative engagement
- However, organizers determined the 2022 workshop duration and content volume were appropriate based on overall satisfaction data (4.49 ± 0.55), completion rates (96.1%), and achievement of learning objectives
- Future consideration: While the cognitive demand is inherent to effective simulation-based learning, consider optimizing pacing or incorporating more frequent breaks

## Key Findings

### 1. Continued High Satisfaction with Multiprofessional Team Format

The multiprofessional team format continued to be successful, with the case-based simulation further enhancing the learning experience.

### 2. Success of Pre-learning Modifications

The combination of streamlined pre-learning and systematic reminder system successfully addressed time burden while maintaining content quality. The dramatic reduction in volume perception (60.5% → 32.5%) validates the differentiation strategy.

### 3. Mobile Optimization Impact

Mobile optimization successfully improved accessibility for the majority of participants who prefer mobile learning. However, platform user experience remains an area for potential refinement.

### 4. Case-Based Simulation Value

Case-based simulation successfully addressed the collaborative practice skills gap identified in 2021, moving beyond knowledge transfer to skill development and practice.

## Implications for Implementation Phase (2023-2024)

Based on refinement phase success, the following components were finalized for broader implementation:

### Core Program Components (Maintained)

1. Multiprofessional team composition: Hospital-based teams from same institution
2. Flipped classroom format: Streamlined pre-learning + interactive workshop
3. Case-based simulation: Structured practice of collaborative skills
4. Multiprofessional faculty: Diverse faculty modeling interprofessional collaboration

### Support Systems (Maintained)

1. Weekly reminder system: Automated emails with direct access links
2. Mobile-optimized platform: Responsive design for smartphone/tablet access
3. Streamlined content: Profession-appropriate pre-learning depth

### Areas for Monitoring

1. Platform user experience: Continue gathering feedback for potential enhancements
2. Pre-learning completion rates: Monitor effectiveness of current timeline and support systems3. Long-term outcomes: Assess behavior change and multidisciplinary team establishment at 6-month follow-up

The program was considered ready for implementation phase while maintaining commitment to ongoing evaluation and continuous quality improvement.

# Supplementary 4: Evolution and Mapping of Competencies, Domains, and Educational Modules (2019–2022)

This supplementary material documents the iterative development of learning objectives and educational content across three action research phases. The framework evolved from faculty consensus (KJ method) to evidence-based competencies (scoping review), with progressive refinement of educational modules aligned with clinical workflow.

## 2019–2020: Initial Framework (Exploratory Phase)

Development Method: KJ method (affinity diagramming) by faculty team
Target Learners: Physicians only
Competency Organization: 7 domains, 22 competencies

| Competencies of Aspiration Pneumonia | Domain | Educational Module |
| --- | --- | --- |
| Appropriate antibiotic treatment | Treatment | Treatment |
| Fluid and electrolyte management |  |  |
| Airway, breathing, and sputum management |  |  |
| Early initiation of oral intake (avoid unnecessary fasting) | Nutrition | Nutrition |
| Modification of diet and liquids according to swallowing function |  |  |
| Evaluation of nutritional status and intake |  |  |
| Supplementation of inadequate oral intake |  |  |
| Appropriate use of tube feeding and prevention of complications |  |  |
| Evaluation of oral status (OHAT, ROAG) | Oral management | Oral management |
| Frequent oral hygiene |  |  |
| Availability of guidance and resources for oral hygiene on the ward |  |  |
| Appropriate use and cleaning of dentures |  |  |
| Dental treatment |  |  |
| Understand the roles of therapists involved in swallowing rehabilitation | Rehabilitation | Rehabilitation |
| Set goals based on the ICF framework and initiate early rehabilitation interventions |  |  |
| Evaluate oral health using standardized tools such as OHAT | Oral/dental assessment and intervention | Oral/dental assessment and intervention |
| Provide instruction and hands-on guidance for oral hygiene |  |  |
| Collaborate effectively with dentists and dental hygienists as needed |  |  |
| Discussions regarding difficult decision making | Multidisciplinary team | Multidisciplinary team |
| Developing protocols for management and risk feeding |  |  |
| Comprehensive team intervention |  |  |
| Providing efficient training for healthcare professionals |  |  |
| Demonstrate one's professional expertise while collaborating with other healthcare professionals | Multidisciplinary collaboration | Multidisciplinary collaboration |
| Share information across disciplines and institutions to provide integrated patient management |  |  |

OHAT: Oral Health Assessment Tool; ROAG: Revised Oral Assessment Guide; ICF: International Classification of Functioning, Disability and Health

## 2021: Reorganization (Prototyping Phase)

Development Method: Transition toward evidence-based framework
Target Learners: Interprofessional teams (physicians, dentists, nurses, pharmacists, therapists, dietitians)
Competency Organization: Reorganized into Knowledge, Skills, Attitude categories
Key Change: Introduction of ’Medication‘ domain

| Competencies of Aspiration Pneumonia | Domain | Educational Module |
| --- | --- | --- |
| Diagnose aspiration pneumonia by integrating findings from swallowing function, aspiration risk, clinical signs, and imaging results | Diagnosis and treatment | Knowledge |
| Identify and intervene on underlying causes or diseases contributing to aspiration pneumonia, such as medication, dementia, or neurodegenerative disorders |  |  |
| Select appropriate antibiotics and determine treatment duration |  |  |
| Evaluate complications associated with aspiration pneumonia |  |  |
| Identify medications that can cause loss of appetite | Medication |  |
| Identify medications that increase the risk of aspiration pneumonia |  |  |
| Identify medications that help prevent aspiration pneumonia |  |  |
| Understand the relationship between drug formulation and swallowing function |  |  |
| Assess nutritional status and evaluate for sarcopenia | Nutrition assessment and guidance |  |
| Estimate energy requirements and provide nutritional guidance according to swallowing ability and food texture |  |  |
| Explain advantages and disadvantages of alternative nutrition methods |  |  |
| Determine indications and select appropriate enteral feeding methods |  |  |
| Perform basic bedside swallowing assessment | Swallow assessment | Skills |
| Determine indications for specialized swallowing evaluations using videoendoscopy, videofluoroscopy, or ultrasound |  |  |
| Select appropriate diet form and initiate early oral feeding based on the initial assessment |  |  |
| Determine optimal positioning for safe swallowing |  |  |
| Understand the roles of therapists involved in swallowing rehabilitation | Rehabilitation |  |
| Set goals based on the ICF framework and initiate early rehabilitation interventions |  |  |
| Evaluate oral health using standardized tools such as OHAT | Oral/dental assessment and intervention |  |
| Provide instruction and hands-on guidance for oral hygiene |  |  |
| Collaborate effectively with dentists and dental hygienists as needed |  |  |
| Demonstrate one's professional expertise while collaborating with other healthcare professionals | Multidisciplinary collaboration | Attitude |
| Share information across disciplines and institutions to provide integrated patient care |  |  |

ICF: International Classification of Functioning, Disability and Health; OHAT: Oral Health Assessment Tool

## 2022: Scoping Review & Finalization (Refinement Phase)

Development Method: Scoping review of 99 studies
Target Learners: Interprofessional teams (expanded to include nurse practitioners, speech and language therapists, dental hygienists, social workers)
Competency Organization: 12 evidence-based domains organized into 8 clinical workflow-aligned modules
Key Changes:
- Evidence-based competency framework
- Introduction of Prevention, Prognosis, and Palliative care domains
- Integration of ethical decision-making
- Module structure aligned with patient care trajectory

| Competencies of Aspiration Pneumonia | Domain | Educational Module |
| --- | --- | --- |
| Diagnose aspiration pneumonia by integrating findings from swallowing function, aspiration risk, clinical signs, and imaging results | Diagnosis | Diagnosis and treatment |
| Diagnostic criteria: presence of aspiration risk factors, clinical signs or symptoms of pneumonia, and radiographic findings in dependent pulmonary segments |  |  |
| Knowing characteristics of aspiration pneumonia and differences from CAP, HAP, HCAP, NHCAP |  |  |
| Detecting pathogens and assessing risk of resistant bacteria |  |  |
| Evaluating X-ray and CT images |  |  |
| Diagnosing underlying condition causing dysphagia/aspiration |  |  |
| Recording and coding correctly in medical records |  |  |
| Identify and intervene on underlying causes or diseases contributing to aspiration pneumonia, such as medication, dementia, or neurodegenerative disorders | Treatment |  |
| Select appropriate antibiotics and determine treatment duration |  |  |
| Evaluate complications associated with aspiration pneumonia |  |  |
| Perform basic bedside swallowing assessment | Swallow assessment | Swallow assessment |
| Determine indications for specialized swallowing evaluations using videoendoscopy, videofluoroscopy, or ultrasound |  |  |
| Select appropriate diet form and initiate early oral feeding based on the initial assessment |  |  |
| Determine optimal positioning for safe swallowing |  |  |
| Early consultation with a speech and language therapist |  |  |
| Observation of eating and drinking habits |  |  |
| Assessment with videofluoroscopy or videoendoscopy |  |  |
| Assessment of silent aspiration and cough effectiveness |  |  |
| Appropriate diet/liquid modification and feeding techniques |  |  |
| Management of dysphagia/aspiration related to underlying conditions | Underlying condition management | Underlying condition management |
| Drug review |  |  |
| Evaluate oral health using standardized tools such as OHAT | Oral management | Oral management |
| Provide instruction and hands-on guidance for oral hygiene |  |  |
| Collaborate effectively with dentists and dental hygienists as needed |  |  |
| Oral hygiene |  |  |
| Understand the roles of therapists involved in swallowing rehabilitation | Rehabilitation | Rehabilitation |
| Set goals based on the ICF framework and initiate early rehabilitation interventions |  |  |
| Early initiation of physical therapy and swallow training |  |  |
| Maintenance of daily activities |  |  |
| Utilization of devices (e.g., TESS) |  |  |
| Assess nutritional status and evaluate for sarcopenia | Nutrition assessment and guidance | Nutrition assessment and guidance |
| Estimate energy requirements and provide nutritional guidance according to swallowing ability and food texture |  |  |
| Explain advantages and disadvantages of alternative nutrition methods |  |  |
| Determine indications and select appropriate enteral feeding methods |  |  |
| Demonstrate one's professional expertise while collaborating with other healthcare professionals | Multidisciplinary team | Multidisciplinary team |
| Share information across disciplines and institutions to provide integrated patient care |  |  |
| Discussions regarding difficult decision making |  |  |
| Developing protocols for aspiration pneumonia management and risk feeding |  |  |
| Comprehensive team intervention |  |  |
| Providing efficient training for healthcare professionals |  |  |
| Oral hygiene | Prevention |  |
| Vaccination |  |  |
| Drug review (discontinuation of risky drugs, appropriate use of preventive drugs) |  |  |
| Acknowledging the complexity of decision making | Decision making | Decision making and palliative care |
| Decisions on administering, withholding, or withdrawing antibiotics, hydration, or resuscitation |  |  |
| Accepting aspiration pneumonia as a natural course of decline in terminal cases |  |  |
| Understanding ethical challenges and dilemmas of difficult decision making |  |  |
| Awareness of poor prognosis compared to non-aspiration pneumonia | Prognosis |  |
| Evaluation of severity, prognosis, and survival rate |  |  |
| Utilization of prognostic factors |  |  |
| Communicating prognosis to patients and families |  |  |
| Subjective and objective symptom assessment | Palliative care |  |
| Symptom control (discomfort, pain, dyspnea, cough, rattling breath) |  |  |

CAP: community acquired pneumonia; HAP: hospital acquired pneumonia; HCAP: Healthcare-associated Pneumonia; NHCAP: Nursing and healthcare-associated pneumonia; CT: computed tomography; OHAT Oral Health Assessment Tool; ICF: International Classification of Functioning, Disability and Health; TESS: Transcutaneous Electrical Sensory Stimulation

**References**

1.	Mandell LA, Niederman MS. Aspiration Pneumonia. N Engl J Med. 2019;380(7):651-63.

2.	Almirall J, Boixeda R, de la Torre MC, Torres A. Epidemiology and Pathogenesis of Aspiration Pneumonia. Semin Respir Crit Care Med. 2024;45(6):621-5.

3.	Tomonaga I, Koseki H, Imai C, Shida T, Nishiyama Y, Yoshida D, et al. Incidence and characteristics of aspiration pneumonia in the Nagasaki Prefecture from 2005 to 2019. BMC Pulm Med. 2024;24(1):191.

4.	Yoshimatsu Y, Aga M, Komiya K, Haranaga S, Numata Y, Miki M, et al. The Clinical Significance of Anaerobic Coverage in the Antibiotic Treatment of Aspiration Pneumonia: A Systematic Review and Meta-Analysis. J Clin Med. 2023;12(5).

5.	Bartlett JG. How important are anaerobic bacteria in aspiration pneumonia: when should they be treated and what is optimal therapy. Infect Dis Clin North Am. 2013;27(1):149-55.

6.	Bartlett JG, Gorbach SL, Finegold SM. The bacteriology of aspiration pneumonia. The American Journal of Medicine. 1974;56(2):202-7.

7.	Marin-Corral J, Pascual-Guardia S, Amati F, Aliberti S, Masclans JR, Soni N, et al. Aspiration Risk Factors, Microbiology, and Empiric Antibiotics for Patients Hospitalized With Community-Acquired Pneumonia. Chest. 2021;159(1):58-72.

8.	Rodriguez AE, Restrepo MI. New perspectives in aspiration community acquired Pneumonia. Expert Rev Clin Pharmacol. 2019;12(10):991-1002.

9.	Yoshimatsu Y, Tobino K, Ko Y, Yasuda M, Ide H, Oku Y. Careful history taking detects initially unknown underlying causes of aspiration pneumonia. Geriatr Gerontol Int. 2020;20(8):785-90.

10.	Smithard DG, Yoshimatsu Y. Pneumonia, Aspiration Pneumonia, or Frailty-Associated Pneumonia? Geriatrics (Basel). 2022;7(5).

11.	Yoshimatsu Y, Melgaard D, Westergren A, Skrubbeltrang C, Smithard DG. The diagnosis of aspiration pneumonia in older persons: a systematic review. Eur Geriatr Med. 2022.

12.	Teramoto S, Fukuchi Y, Sasaki H, Sato K, Sekizawa K, Matsuse T, et al. High incidence of aspiration pneumonia in community- and hospital-acquired pneumonia in hospitalized patients: a multicenter, prospective study in Japan. J Am Geriatr Soc. 2008;56(3):577-9.

13.	The Japanese Respiratory Society. **The JRS Guidelines for the Management of Pneumonia in Adults 2024**

Japan: Medical Review Co; 2024.

14.	Almirall J, Yoshimatsu Y, FA S. Respiratory infections and aspiration pneumonia. In: Clavé P OO, editor. A Multidisciplinary Approach to Managing Swallowing Dysfunction in Older People: Academic Press, Elsevier; 2024. p. 169–77.

15.	Yoshimatsu Y, Thomas H, Thompson T, Smithard DG. Prognostic factors of poor outcomes in pneumonia in older adults: aspiration or frailty? European Geriatric Medicine. 2024.

16.	Ekberg O, Hamdy S, Woisard V, Wuttge-Hannig A, Ortega P. Social and psychological burden of dysphagia: its impact on diagnosis and treatment. Dysphagia. 2002;17(2):139-46.

17.	Simpson AJ, Allen JL, Chatwin M, Crawford H, Elverson J, Ewan V, et al. BTS clinical statement on aspiration pneumonia. Thorax. 2023;78(Suppl 1):s3-s21.

18.	Lesa L, Brunelli L, Valent F, Aprili I, Cametti E, Ferrari S, et al. The underestimated burden of aspiration event and pneumonia within hospitals: what happens after dysphagia. Ann Ig. 2021;33(6):555-63.

19.	Yoshimatsu Y, Markowski M, Smithard DG, Hansjee D, Hashimoto T, Nagano H, et al. Eating and Drinking with Acknowledged Risks (EDAR) in Older Adults: A Qualitative Study of the Experiences of Clinicians in Japan and the UK. Dysphagia. 2024.

20.	Yoshimatsu Y, Ohtake Y, Ukai M, Miyagami T, Morikawa T, Shimamura Y, et al. "Diagnose, Treat, and SUPPORT". Clinical competencies in the management of older adults with aspiration pneumonia: a scoping review. Eur Geriatr Med. 2023.

21.	Baijens LW, Clave P, Cras P, Ekberg O, Forster A, Kolb GF, et al. European Society for Swallowing Disorders - European Union Geriatric Medicine Society white paper: oropharyngeal dysphagia as a geriatric syndrome. Clin Interv Aging. 2016;11:1403-28.

22.	Yoshimatsu Y, Smithard DG. A Paradigm Shift in the Diagnosis of Aspiration Pneumonia in Older Adults. J Clin Med. 2022;11(17).

23.	Ueda A, Nohara K. Global trends in aspiration pneumonia research since 1980: A bibliometric analysis. Medicine (Baltimore). 2025;104(23):e42808.

24.	Lingard L, Albert M, Levinson W. Grounded theory, mixed methods, and action research. BMJ. 2008;337:a567.

25.	Koshy E, Koshy V, Waterman H. Action research in healthcare. London: SAGE; 2011.

26.	Cohen L, Manion L, K M. Research methods in education. 6th ed. London and New York: Routledge.

27.	Smith L, Rosenzweig L, Schmidt M. Best Practices in the Reporting of Participatory Action Research: Embracing Both the Forest and the Trees 1Ψ7. The Counseling Psychologist. 2010;38(8):1115-38.

28.	Ogrinc G, Davies L, Goodman D, Batalden P, Davidoff F, Stevens D. SQUIRE 2.0 (Standards for QUality Improvement Reporting Excellence): revised publication guidelines from a detailed consensus process. BMJ Qual Saf. 2016;25(12):986-92.

29.	Meyer J. Evaluating action research. Age Ageing. 2000;29 Suppl 2:8-10.

30.	Kern DE, Thomas PA, Howard DM, EB B. Curriculum Development for Medical Education: A Six-Step Approach. Baltimore, MD: Johns Hopkins University Press; 1998.

31.	Wenger E. Communities of Practice: Learning, Meaning, and Identity. Cambridge: Cambridge University Press; 1998.

32.	Lave J, Wenger E. Situated Learning: Legitimate Peripheral Participation. 1991.

33.	Mezirow J. Transformative Learning: Theory to Practice. New Directions for Adult and Continuing Education. 2002;1997(74):5-12.

34.	McLaughlin JE, Roth MT, Glatt DM, Gharkholonarehe N, Davidson CA, Griffin LM, et al. The flipped classroom: a course redesign to foster learning and engagement in a health professions school. Acad Med. 2014;89(2):236-43.

35.	Scupin R. The KJ Method: A Technique for Analyzing Data Derived from Japanese Ethnology. Human Organization.56:233–37.

36.	World Health Organization. International Classification of Functioning, Disability and Health (ICF) Geneva: WHO; 2001 [Available from: <https://apps.who.int/iris/bitstream/handle/10665/42407/9241545429.pdf>.

37.	Jonsen AR, Siegler M, Winslade WJ. Clinical ethics: a practical approach to ethical decisions in clinical medicine. Siegler M, WJ W, editors. New York: McGraw Hill; 2022.

38.	Chalmers JM, King PL, Spencer AJ, Wright FA, Carter KD. The oral health assessment tool--validity and reliability. Aust Dent J. 2005;50(3):191-9.

39.	Kirkpatrick DL, JD K. Evaluating Training Programs: The Four Levels.: Berrett-Koehler Publishers; 2006.

40.	Yardley S, Dornan T. Kirkpatrick's levels and education 'evidence'. Med Educ. 2012;46(1):97-106.

41.	Star SL, Griesemer JR. Institutional Ecology, `Translations' and Boundary Objects: Amateurs and Professionals in Berkeley's Museum of Vertebrate Zoology, 1907-39. Social Studies of Science. 1989;19(3):387-420.

42.	Liu Q, Peng W, Zhang F, Hu R, Li Y, Yan W. The Effectiveness of Blended Learning in Health Professions: Systematic Review and Meta-Analysis. J Med Internet Res. 2016;18(1):e2.

43.	Vallee A, Blacher J, Cariou A, Sorbets E. Blended Learning Compared to Traditional Learning in Medical Education: Systematic Review and Meta-Analysis. J Med Internet Res. 2020;22(8):e16504.

44.	Masters K, Ellaway RH, Topps D, Archibald D, Hogue RJ. Mobile technologies in medical education: AMEE Guide No. 105. Med Teach. 2016;38(6):537-49.

45.	Yoshimatsu Y, Hansjee D, Markowski M, Essex R, Smithard DG. Decisions on eating and drinking in older adults admitted with pneumonia and referred for swallowing difficulties. Eur Geriatr Med. 2024;15(3):771-5.

46.	Jørgensen R, Scarso E, Edwards K, Ipsen C. Communities of practice in healthcare: A framework for managing knowledge sharing in operations. Knowledge and Process Management. 2018;26(2):152-62.

47.	Fingrut W, Beck LA, Lo D. Building an oncology community of practice to improve cancer care. Curr Oncol. 2018;25(6):371-7.

48.	Hamoen EC, van Blankenstein FM, de Jong PGM, Ray A, Reinders MEJ. Development of a Clinical Teaching Unit in Internal Medicine to Promote Interprofessional and Multidisciplinary Learning: A Practical Intervention. Teach Learn Med. 2021;33(1):78-88.

49.	Miller D, Kwan D, Ng S, Friesen F, Lowe M, Maniate J, et al. Initiating communities of practice for teaching and education scholarship in hospital settings: a multi-site case study. MedEdPublish (2016). 2018;7:127.

50.	Sahay A, Mittman BS, Gholami P, Lin S, Heidenreich PA. How successful was the use of a community of practice for the implementation of evidence-based practices for heart failure within the United States Department of Veterans Affairs: Insights from a formative evaluation. Health Res Policy Syst. 2022;20(1):79.

51.	Sahay A, Mittman BS, Gholami P, Lin S, Heidenreich PA. Use of a Community of Practice for the Implementation of Evidence-based Practices for Heart Failure Within the United States Department of Veterans Affairs. Unknown Journal. 2021.

52.	Li C-H, Su K-S, Liu S-F. Leadership Style and the Internal Processes in Communities of Practices. Universal Journal of Management. 2015;3(10):407-12.

53.	Ikegami N, Campbell JC. Medical care in Japan. N Engl J Med. 1995;333(19):1295-9.

54.	Katori T. Japan's healthcare delivery system: From its historical evolution to the challenges of a super-aged society. Glob Health Med. 2024;6(1):6-12.

55.	Tun TZ, Thwin KM, Takehara S, Minagawa K, Kaneko N, Ogawa H. Impacts of dental checkups on oral function among Japanese older adults. Sci Rep. 2025;15(1):17622.
